# The effect of nicotine smoking on cognitive performance in patients with schizophrenia

**DOI:** 10.1192/j.eurpsy.2026.12163

**Published:** 2026-07-31

**Authors:** L. Kalisova, K. Poshor, J. Michalec, F. Dechterenko

**Affiliations:** 1 Department of Psychiatry and Neuropsychiatry, 2nd Faculty of Medicine of Charles University and Military Hospital; 2 Department of Psychology, Philosophical Faculty of Charles University; 3 Institute of Psychology, Czech Academy of Sciences, Prague, Czech Republic

## Abstract

**Introduction:**

The prevalence of nicotine smoking in schizophrenia patients is 2-3 times higher than in the general population. As high as 45-80% of schizophrenia patients smoke. Although the number of smokers has decreased in many European countries in the last decade, the number of smokers with schizophrenia remains stationary. Smoking is associated with higher mortality and morbidity. Patients with schizophrenia make few attempts to quit smoking and are less aware of the risks of smoking. The reasons for smoking in schizophrenia patients are various, from genetic predisposition to self-medication to reduce the side effects of medication, and also, social factors can play a role. Nicotine can improve cognitive performance in the short term by acting on nicotinic receptors in the brain. Long-term smoking can negatively affect cognitive performance.

**Objectives:**

The presented study aimed to evaluate the cognitive performance of smokers and non-smokers with a diagnosis of schizophrenia, divided by duration of illness /smoking.

**Methods:**

To assess cognitive functions, we used the MCCB (MATRICS Consensus Cognitive Battery), excluding the social cognition domain. To estimate premorbide intellectual capacity NART (National Adult Reading Test) was used. Basic sociodemographic characteristics (age, gender, and education) and information on the duration of illness were also gathered.

**Results:**

A Sample of 184 patients with schizophrenia was divided into two groups according to smoking status. We included 102 smokers (age: 34 ± 10 years, 19% female) and 82 non-smokers (age: 35 ± 10 years, 18% female). 31% of the sample had a FES (first episode of schizophrenia; first two years of clearly expressed symptoms of schizophrenia), 69% longer-term course of the illness. Non-smokers have higher levels of education and NART in comparison with smokers. FES smokers have similar and, in some domains, even better results in MCCB cognitive testing compared to FES non-smokers. Longer course schizophrenia patients - smokers had significantly worse results in MCCB in comparison with FES smokers, and also non-smokers.

**Conclusions:**

Chronic smoking has a clearly negative effect on health, including mental health and cognitive performance of patients with schizophrenia. Treatment of schizophrenia should also include education about the risk of nicotine smoking and motivation for smoking cessation.

**Disclosure of Interest:**

None Declared

